# CD3D Is an Independent Prognostic Factor and Correlates With Immune Infiltration in Gastric Cancer

**DOI:** 10.3389/fonc.2022.913670

**Published:** 2022-06-01

**Authors:** Li Yuan, Jingli Xu, Yunfu Shi, Zhiyuan Jin, Zhehan Bao, Pengcheng Yu, Yi Wang, Yuhang Xia, Jiangjiang Qin, Bo Zhang, Qinghua Yao

**Affiliations:** ^1^The Cancer Hospital of the University of Chinese Academy of Sciences (Zhejiang Cancer Hospital), Institutes of Basic Medicine and Cancer (IBMC), Chinese Academy of Sciences, Hangzhou, China; ^2^Zhejiang Provincial Research Center for Upper Gastrointestinal Tract Cancer, Zhejiang Cancer Hospital, Hangzhou, China; ^3^Zhejiang Key Laboratory of Prevention, Diagnosis and Therapy of Upper Gastrointestinal Cancer, Zhejiang Cancer Hospital, Hangzhou, China; ^4^First Clinical Medical College, Zhejiang Chinese Medical University, Hangzhou, China; ^5^Department of Integrated Chinese and Western Medicine, Institute of Basic Medicine and Cancer (IBMC), The Cancer Hospital of the University of Chinese Academy of Sciences (Zhejiang Cancer Hospital), Chinese Academy of Sciences, Hangzhou, China; ^6^Key Laboratory of Traditional Chinese Medicine Oncology, Zhejiang Cancer Hospital, Hangzhou, China; ^7^Key Laboratory of Head and Neck Cancer Translational Research of Zhejiang Province, Hangzhou, China

**Keywords:** gastric cancer, cd3d, tumor infiltrating lymphocytes (TILs), prognosis, PD-L1

## Abstract

The protein encoded by CD3D is part of the T-cell receptor/CD3 complex (TCR/CD3 complex) and is involved in T-cell development and signal transduction. Previous studies have shown that CD3D is associated with prognosis and treatment response in breast, colorectal, and liver cancer. However, the expression and clinical significance of CD3D in gastric cancer are not clear. In this study, we collected 488 gastric cancer tissues and 430 paired adjacent tissues to perform tissue microarrays (TMAs). Then, immunohistochemical staining of CD3D, CD3, CD4, CD8 and PD-L1 was conducted to investigate the expression of CD3D in gastric cancer and the correlation between the expression of CD3D and tumor infiltrating lymphocytes (TILs) and PD-L1. The results showed that CD3D was highly expressed in gastric cancer tissues compared with paracancerous tissues (*P*<0.000). Univariate and multivariate analyses showed that CD3D was an independent good prognostic factor for gastric cancer (*P*=0.004, HR=0.677, 95%CI: 0.510-0.898 for univariate analyses; *P*=0.046, HR=0.687, 95%CI: 0.474-0.994 for multivariate analyses). In addition, CD3D was negatively correlated with the tumor location, Borrmann type and distant metastasis (*P*=0.012 for tumor location; *P*=0.007 for Borrmann type; *P*=0.027 for distant metastasis). In addition, the expression of CD3D was highly positively correlated with the expression of CD3, CD4, CD8, and PD-L1, and the combination of CD3D with CD3, CD4, CD8 and PD-L1 predicted the best prognosis (*P*=0.043). In summary, CD3D may play an important regulatory role in the tumor immune microenvironment of gastric cancer and may serve as a potential indicator of prognosis and immunotherapy response.

## Introduction

Gastric cancer is one of the most common malignant tumors in the world. According to the latest data, there are more than 1 million new cases of gastric cancer in the world, and approximately 770,000 people have died of gastric cancer. Mortality and morbidity rank 4th and 5th, respectively, and seriously threaten human life and health (1). At present, the diagnosis of gastric cancer mainly relies on endoscopy and tissue biopsy. However, due to its invasiveness and high cost, more than 60% of patients with gastric cancer are in the middle or advanced stage at the time of diagnosis, and the prognosis is poor (2). At present, surgery is the main treatment for early gastric cancer, and comprehensive treatments such as surgery, radiotherapy and chemotherapy, targeted therapy, and immunotherapy are the main strategies to combat advanced gastric cancer. However, due to the lack of therapeutic targets and the high tumor heterogeneity of gastric cancer, the population of patients who benefit from targeted therapy and immunotherapy is limited. Therefore, it is urgent to improve the level of early diagnosis and identify new therapeutic targets.

The protein product of the CD3D gene is part of the T-cell receptor/CD3 complex (TCR/CD3 complex) and is involved in T-cell development and signal transduction (3). The integrity of the TCR/CD3 complex is crucial for the effector and regulatory functions of peripheral T lymphocytes (4). An increasing number of studies have shown that CD3D is closely related to the occurrence, development, prognosis, immune microenvironment and treatment response of tumors. Chen et al. have found that CD3D was significantly negatively correlated with PD1 and could predict immunochemotherapy response in diffuse large B-cell lymphoma (5). Kang et al. have demonstrated that CD3D expression reduced the prognostic risk of bladder cancer (6). Saiz-Ladera *et al.* have confirmed that CD2, CD3D, CD3E and CXCR6 combined gene expression was associated with an improvement in the outcomes of head and neck squamous cell carcinoma patients and an increase in infiltrating immune effector cells (7). However, the expression and clinical significance of CD3D in gastric cancer are still unclear and are worthy of further study.

In this study, we collected 488 gastric cancer tissues and 430 paired adjacent tissues to explore the difference in CD3D expression between tumor tissues and paracancerous tissues, as well as the correlation between the expression of CD3D and clinical information, prognosis, tumor-infiltrating lymph nodes (CD3+ T cells, CD4+ T cells and CD8+ T cells) and PD-L1. The aim of this study was to clarify the value of CD3D in the diagnosis and prognosis of gastric cancer and its role in the tumor immune microenvironment.

## Materials and Methods

### Clinical Specimens

We retrospectively collected 488 patients with gastric cancer treated at The Affiliated Cancer Hospital of the University of Chinese Academy of Sciences (Zhejiang Cancer Hospital) from January 2013 to December 2017. The inclusion criteria were as follows: patients with gastric cancer confirmed by pathological diagnosis; and no prior antitumor treatment, such as chemoradiotherapy, targeted therapy and immunotherapy. The exclusion criteria were as follows: patients with gastric cancer and other types of malignant tumors at the same time; patients with metastasis from other tumor species to the stomach; and patients who received prior antitumor treatment. Overall survival (OS) was defined as the duration from the initial surgery to the date of death or the last follow-up.

All clinical information of the participants was collected, including age, gender, height, weight, family history, smoking status, alcohol consumption, TNM staging, blood tumor markers, etc. Pathological staging was based on the American Joint Committee on Cancer 8th edition staging (8). The study was approved by the Research Ethics Committee of the Zhejiang Cancer Hospital (IRB-2021-431).

### Tissue Microarray (TMA) Construction and Immunohistochemistry Analysis

We collected 488 formalin-fixed, paraffin-embedded gastric cancer tissue specimens and 430 corresponding adjacent noncancerous tissue specimens. Two pathologists independently selected the most representative tumors and paired adjacent tissues, and TMAs were performed as described previously (9). For immunohistochemistry assays, after treatment with 3% H2O2/methyl alcohol solution for 10 min at room temperature, 5% normal goat serum buffer was used to block the tissue at 37 °C for 30 min. Slides were then incubated with primary antibodies at 4°C overnight. After washing, the slides were incubated with biotin-labeled goat anti-rabbit IgG and HRP-conjugated streptavidin at 37°C for 1 h. Immunoreaction was visualized by diaminobenzidine (DAB) (Cat# ZLI-9065, ZSGB-BIO Corp., Shanghai, China). After DAB staining, all tissues were counterstained with hematoxylin (Cat# ZLI-9609 ZSGB-BIO Corp., Shanghai, China), dehydrated and then blocked. The antibodies against CD3D (Cat# 109531), CD3 (Cat# 16669), CD4 (Cat# 133616), and CD8 (Cat# 17147) were purchased from Abcam (Cambridge, UK). PD-L1 (Cat# SK006) was purchased from Dako Denmark A/S (Copenhagen, Denmark).

The immunohistochemical staining results were interpreted by two experienced pathologists. The expression of PD-L1 was evaluated by the combined positive score (CPS) score (CPS=PD-L1-positive cells (tumor cells, lymphocytes, macrophages)/total tumor cells). The CPS evaluation criteria were as follows: tumor cells with membrane staining at any intensity directly related to tumor cells, and membrane/cytoplasmic staining of lymphocytes/macrophages. These stained cells accounted for a percentage of the total tumor cells. The stained cells should exclude all necrotic cells, mesenchymal cells, carcinoma *in situ* and other immune cells (including but not limited to neutrophils, eosinophils and plasma cells). The expression of CD3D, CD3, CD4, and CD8 was divided into a high-expression group and a low-expression group according to the median number of positive cells (10, 11). The median numbers of CD3D-, CD3-, CD4-, and CD8-positive cells were 120, 190, 30 and 110, respectively.

### Statistical Analysis

SPSS 23.0 software was used for statistical analysis of all data, and the measurement data are expressed as the median ± standard error. GraphPad Prism 8.3.0 was used for mapping. The relationship between CD3D expression and clinicopathological features, tumor-infiltrating lymphocytes and PD-L1 expression was analyzed by the chi-square test or Fisher’s exact test. Kaplan–Meier analysis was used to draw survival curves, and univariate and multivariate Cox regression analyses were used to determine the prognostic factors of gastric cancer. *P*<0.05 indicates a significant difference.

## Results

### Characteristics of the Participants

A total of 488 gastric cancer patients were recruited, ranging in age from 28 to 91, with an average age of 64.05 ± 0.48, including 360 males (73.77%) and 128 females (26.23%), which is consistent with the epidemiological situation of gastric cancer, indicating that the included cases were generally representative. In terms of TNM staging, 22 patients had stage I, 70 patients had stage II, 357 patients had stage III, and 32 patients had stage IV disease. In terms of the pathological types, gastric adenocarcinoma was the main type, accounting for 454 cases (93.03%), and only 34 (6.97%) were of other types. More detailed information is shown in **Table 1** and **Table 1S in Supplementary Material**.

**Table 1:** Clinicopathological features of 488 patients with gastric cancer.

Clinicopathological features	Number (%)/Mean ± standard error
Age (year)	64 (28, 91) 64.05 ± 0.48
Gender	
Male/Female	360/128 (73.77%/26.23%)
Family history (gastric cancer)	
Yes/No/Unkown	39/318/131 (7.99%/65.16%/26.84%)
Smoking history	
Yes/No/Unkown	105/252/131 (21.52%/51.64%/26.84%)
Drinking history	
Yes/No/Unkown	77/280/131 (15.78%/57.38%/26.84%)
Lose weight	
Yes/No/Unkown	105/251/132 (21.52%/51.43%/27.05%)
Tumor location	
Proximal/Distal/Unkown	246/229/13 (50.41%/46.93%/2.66%)
Borrmann type	
I/II/III/IV	20/208/203/21/136 (4.10%/42.62%/41.60%/4.30%/27.87%)
Lauren type	
Intestinal/Diffuse/Mixed/Unkown	197/108/46/137 (40.37%/22.13%/9.43%/28.07%)
Tumor size (cm)	
>5/≤5/Unkown	233/250/5 (47.75%/51.23%/1.02%)
Grade of differentiation	
Poor/Moderate-poor/Moderate+Well/Unkown	172/100/63/153 (35.25%/20.50%/12.91%/31.35%)
Pathological type	
Adenocarcinoma/Others	454/34 (93.03%/6.97%)
T stage	
1+2/3+4/Unkown	41/396/51 (8.40%/81.15%/10.45%)
N stage	
0/1+2+3/Unkown	77/402/9 (15.78%/82.38%/1.84%)
M stage	
0/1/Unkown	447/34/7 (91.60%/6.97%/1.43%)
TNM stage	
I/II/III/IV	22/70/355/34/7 (4.51%/14.34%/72.75%/6.97%/1.43%)
AFP (ng/ml)	
≤8.1/>8.1/Unkown	309/19/160 (63.32%/3.90%/32.78%)
CEA (ng/ml)	
≤5/>5/Unkown	247/83/158 (50.61%/17.01%/32.38%)
CA199 (U/ml)	
≤37/>37/Unkown	234/96/158 (47.95%/19.67%/32.38%)
CA724 (U/ml)	
≤6.9/>6.9/Unkown	259/54/175 (53.07%/11.07%/35.86%)
CA125 (U/ml)	
≤35/>35/Unkown	280/15/193 (57.38%/3.07%/39.55%)
CA50 (U/ml)	
≤25/>25/Unkown	230/38/220 (47.13%/7.79%/45.08%)

### CD3D Is Highly Expressed in Gastric Cancer Tissues and Predicts a Good Prognosis

**Figure 1:**
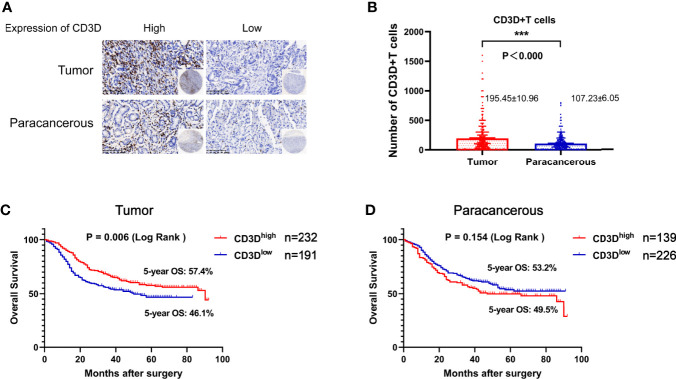
CD3D is overexpressed in gastric cancer tissues compared with adjacent tissues, and a high expression of CD3D is associated with a good prognosis in gastric cancer patients. **(A)** Representative images of CD3D staining in TMAs as determined by immunohistochemical analysis. **(B)** Differential expression of CD3D in gastric cancer tissue and paracancerous tissue. **(C)** The overall survival (OS) curves of gastric cancer patients with different CD3D expression levels in tumor tissues as determined by Kaplan–Meier analysis (log-rank test). **(D)** The OS curves of gastric cancer patients with different CD3D expression levels in paracancerous tissues as determined by Kaplan–Meier analysis (log-rank test). ***p < 0.001.

As shown in **Figure 1A**, CD3D was mainly expressed on lymphocytes. The average number of CD3D-positive cells in the tumor tissues was 195.45 ± 10.96, while that in the paracancerous tissues was 107.23 ± 6.05, indicating a significant difference (**Figure 1B** and **Table 2**, *P*<0.000). Kaplan–Meier survival analysis indicated that the 5-year overall survival rate of patients with high CD3D expression in tumor tissues was 57.4%, while that of patients with low CD3D expression was 46.1%, showing a significant difference (**Figure 1C**, *P*=0.006). We also analyzed the effect of CD3D expression in paracancerous tissues on prognosis, and the results showed that the expression of CD3D in paracancerous tissues was not associated with prognosis (**Figure 1D**, *P*=0.154).

**Table 2:** The differential expression of CD3D in tumor tissues and paracancerous tissues.

Parameters	N	CD3D expression		Number of positive cells (Mean ± standard error)	T	*P* value
		High	Low			
Tumor tissue	488	244	244	195.45 ± 10.96	-7.046	<0.000
Paracancerous tissue	430	146	284	107.23 ± 6.05

### Expression of CD3D Is Correlated With the Tumor Location, Borrmann Type and Distant Metastasis

To further observe the significance of CD3D expression in gastric cancer, we analyzed the correlation between its expression and all clinical information, such as age, gender, smoking status, and alcohol consumption. We found that The expression of CD3D was correlated with tumor location, and is significantly higher in proximal gastric cancer than in distal gastric cancer (**Table 3**, *P*=0.012). Besides, the CD3D positivity rate was 65.63% among patients with Borrmann type I+II but only 49.55% among those with type III+IV, showing a significant difference (**Table 3**, *P*=0.004). In addition, the CD3D positivity rate was 31.25% in gastric cancer patients with distant metastases, while it reached as high as 51.45% in patients without distant metastases, indicating a significant difference (**Table 3**, *P*=0.027). To summarize, the expression of CD3D was found to be significantly reduced in patients with distant metastasis or in patients with Borrmann type III+IV. In addition, the expression of CD3D was not associated with clinical characteristics such as age, gender, height, weight, family history, smoking status, alcohol consumption, tumor size, or serum tumor markers (**Table 3**).

**Table 3:** The correlation between CD3D expression and clinicopathological characteristics in gastric cancer.

Parameters	CD3D expression		Total	Positive rate	χ2	*P* value
	High	Low				
Age(years)						
≥65	113	130	243	46.50%	2.369	0.124
<65	131	114	245	53.47%
Gender						
Female	71	57	128	55.47%	2.076	0.150
Male	173	187	360	48.06%
Family history						
Yes	24	15	39	61.54%	0.715	0.398
No	173	145	318	54.40%
Unkown	131					
Smoking history						
Yes	54	51	105	51.43%	0.847	0.357
No	143	109	252	56.75%
Unkown	131					
Drinking history						
Yes	41	36	77	53.25%	0.149	0.700
No	156	124	280	55.71%
Unkown	131					
Weight loss						
Yes	60	45	105	57.14%	0.196	0.658
No	137	114	251	54.58%
Unkown	132					
Tumor location						
Proximal	104	135	239	43.51%	6.360	**0.012**
Distal	130	106	236	55.08%
Unkown	13					
Borrmann type						
I/II	84	44	128	65.63%	8.515	**0.004**
III/IV	111	113	224	49.55%
Unkown	136					
Lauren type						
Intestinal	110	87	197	55.84%	3.146	0.207
Diffuse	66	42	108	61.11%
Mixed	21	25	46	45.65%
Unkown	137					
Tumor size (cm)						
>5cm	110	123	233	47.21%	1.299	0.254
≤5cm	131	119	250	52.4%
Unkown	5					
Grade of differentiation						
Poor	96	76	172	55.81%	2.475	0.290
Moderate-poor	61	39	100	61.00%
Moderate+Well	30	32	62	48.39%
Unkown	154					
T stage						
T1/2	21	20	41	51.22%	0.009	0.926
T3/4	220	216	436	50.46%
Unkown	11					
N stage						
N0/1	89	79	168	52.98%	0.854	0.356
N2/3	151	160	311	48.55%
Unkown	9					
M stage						
M0	231	218	449	51.45%	4.874	**0.027**
M1	10	22	32	31.25%
Unkown	7					
TNM stage						
I/II	50	42	92	54.35%	0.820	0.365
III/IV	191	198	389	49.10%
Unkown	7					
AFP (ng/ml)						
>8.1	11	8	19	57.89%	0.018	0.892
≤8.1	174	135	309	56.31%
Unkown	160					
CEA (ng/ml)						
>5	44	39	83	53.01%	0.603	0.437
≤5	143	104	247	57.89
Unkown	158					
CA199 (U/ml)						
>37	50	46	96	52.08%	1.158	0.282
≤37	137	97	234	58.55%
Unkown	158					
CA724 (U/ml)						
>6.9	31	23	54	57.41%	0.120	0.729
≤6.9	142	117	259	54.83%
Unkown	175					
CA125 (U/ml)						
>35	12	3	15	80.00%	2.117	0.146
≤35	161	119	280	57.50%
Unkown	193					
CA50 (U/ml)						
>25	17	21	38	44.74%	0.722	0.396
≤25	120	110	230	52.17%
Unkown	220					

The results with statistical differences are bold values.

### CD3D Regulates the Immune Microenvironment of Gastric Cancer

**Figure 2:**
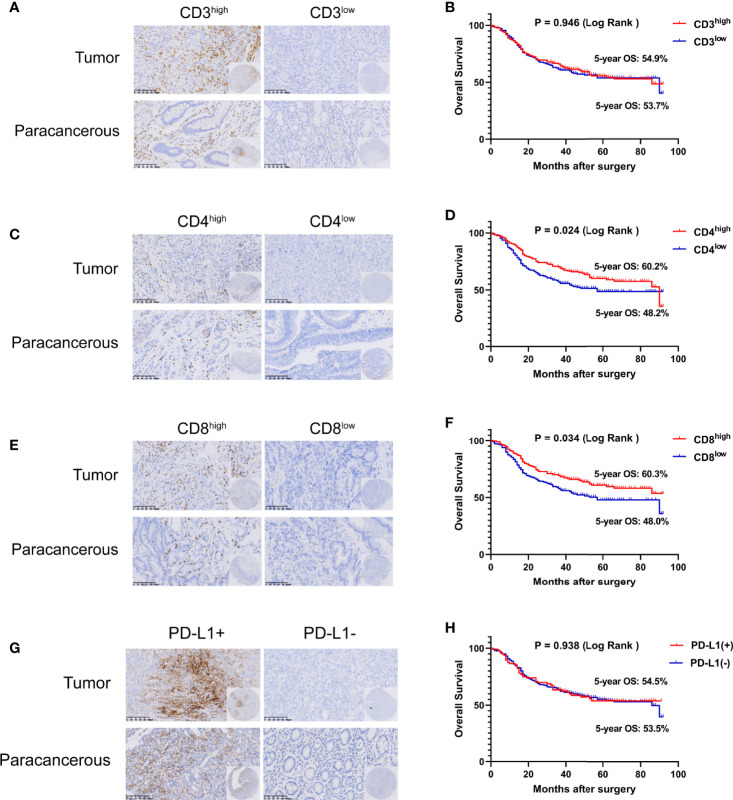
Tumor infiltrating lymphocytes (TILs) and PD-L1 are closely related to the prognosis of gastric cancer. Representative images of CD3 **(A)**, CD4 **(C)**, CD8 **(E)**, and PD-L1 **(G)** staining in TMAs as determined by immunohistochemical analysis. The overall survival (OS) curves of gastric cancer patients with different CD3 **(B)**, CD4 **(D)**, CD8 **(F)**, and PD-L1 **(H)** expression levels in tumor tissues as determined by Kaplan–Meier analysis (log-rank test).

Some studies have shown that CD3D is involved in regulating the tumor immune microenvironment in cervical cancer, liver cancer, breast cancer and other tumors (12–14). Therefore, we further detected the expression of CD3, CD4, CD8 and PD-L1 in the tumor tissues of the included gastric cancer patients and evaluated their relationship with CD3D to explore the regulatory role of CD3D in the immune microenvironment of gastric cancer. We divided the patients into a high-expression group and a low-expression group according to the median number of cells positive for CD3, CD4 and CD8 (**Figures 2A, C, E**). For PD-L1 interpretation, a CPS score greater than or equal to 10 was considered positive, and a CPS score less than 10 was considered negative (15–17) (**Figure 2G**). Kaplan–Meier survival analysis showed that the expression of CD4 and CD8 was correlated with the survival of gastric cancer patients, while the expression of CD3 and PD-L1 was not (**Figures 2B, D, F, H**). The 5-year overall survival rates of the CD4 and CD8 high-expression groups were 60.2% and 60.3%, respectively, while those of the CD4 and CD8 low-expression groups were 48.2% and 48.0%, respectively (**Figure 2D**, *P*=0.024 and **2F**, *P*=0.034).

**Figure 3:**
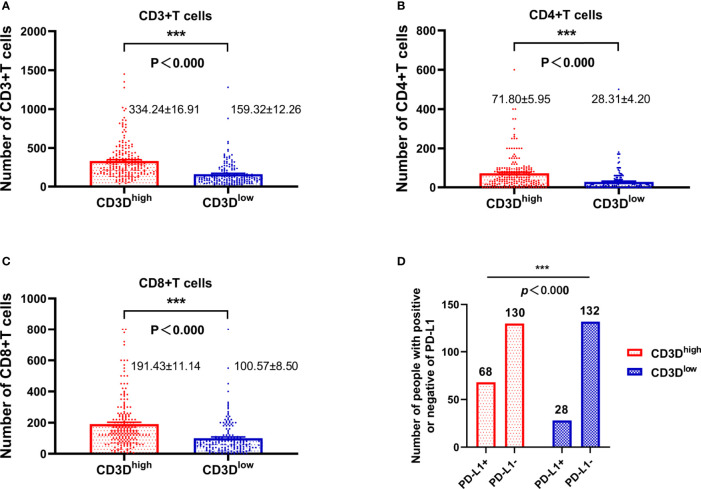
The expression of CD3D is highly positively correlated with the expression of CD3, CD4, CD8, and PD-L1. **(A)** The relationship between the expression of CD3D and CD3. **(B)** The relationship between the expression of CD3D and CD4. **(C)** The relationship between the expression of CD3D and CD8. **(D)** Relationship between the expression of CD3D and PD-L1. ***p < 0.001.

As shown in **Figure 3**, the expression of CD3D was closely related to that of CD3, CD4, CD8 and PD-L1. We found that CD3D was positively correlated with the expression of CD3, CD4, and CD8, and the expression of CD3, CD4, and CD8 in the CD3D high-expression group was higher than that in the CD3D low-expression group, with significant differences observed (**Figures 3A–C**, all *P*<0.000). Again, Pearson correlation coefficients showed the same result (**Table 4**). In addition, we found that CD3D was positively correlated with the expression of PD-L1. The PD-L1 positivity rate was 34.34% in the group with high CD3D expression but only 17.5% in the group with low CD3D expression (**Figure 3D**, *P*<0.000). The Pearson correlation coefficient also found a positive correlation between CD3D and PD-L1 expression (**Table 4**). In conclusion, CD3D may play an important regulatory role in the tumor immune microenvironment by regulating tumor infiltrating lymphocytes (TILs) and immune checkpoints and may be a potential immunotherapy target.

**Table 4:** The pearson correlation coefficients between CD3D expression and CD3, CD4, CD8 and PD-L1 expression in gastric cancer.

Parameters	CD3D vs CD3	CD3D vs CD4	CD3D vs CD8	CD3D vs PD-L1
Pearson Correlation	0.668	0.466	0.623	0.166
*P*	**<0.0001**	**<0.0001**	**<0.0001**	**0.002**
95% CI	0.625-0.723	0.411-0.535	0.584-0.672	0.064-0.277

The results with statistical differences are bold values.

### CD3D^high^ Combined With CD4^high^, CD8 ^high^ and PD-L1- Predicts the Best Prognosis of Gastric Cancer

Risk factors were determined by univariate and multivariate logistic regression analyses. Univariate logistic regression analysis found that CD3D, CD8, CD4, age, family history, tumor location, tumor size, grade of differentiation and TNM stage were all prognostic factors for gastric cancer (**Table 5**). In addition, we further carried out multivariate analysis with these important factors, and the results showed that CD3D, CD4, and CD8 were independent prognostic factors for gastric cancer in both the univariate and multivariate analyses (**Table 6**).

**Table 5:** Univariate Cox regression analysis of overall survival in gastric cancer patients.

Parameters	Univariate COX regression analysis		
	*P* value	HR	(95%CI)
CD3D expression			
High vs Low	**0.007**	0.677	0.510-0.898
Gender			
Female vs. Male	0.963	1.008	0.734-1.384
Age(year)			
≥65 vs <65	**0.035**	1.357	1.022-1.800
Gastric history			
Yes vs No	**<0.0001**	1.444	1.195-1.744
Smoking history			
Yes vs No	**0.006**	1.322	1.082-1.615
Drinking history			
Yes vs No	**0.006**	1.319	1.082-1.607
Weight loss			
Yes vs No	**0.001**	1.391	1.142-1.694
Tumor location			
Proximal vs distal	**0.018**	0.707	0.531-0.943
Pathological type			
Adenocarcinoma/Others	0.634	1.137	0.671-1.925
Borrmann type			
I+II vs III+IV	**0.038**	1.410	1.019-1.950
Lauren type			
Intestinal vs diffuse	0.051	1.349	0.999-1.823
Tumor size (cm)			
≤5 vs.>5	**0.027**	1.378	1.037-1.831
Grade of differentiation			
Poor vs moderate-poor+ Moderate+well	**0.048**	1.387	1.003-1.918
T stage			
T1+T2 vs T3+T4	**0.006**	2.711	1.335-5.502
N stage			
N0+N1 vs N2+3	**<0.0001**	3.208	2.248-4.576
M stage			
M0 vs M1	**<0.0001**	3.529	2.289-5.439
TNM stage			
I+II vs III+IV	**<0.0001**	2.436	1.576-3.764
AFP(ng/ml)			
≤8.1 vs >8.1	**<0.0001**	1.754	1.278-2.408
CEA(ng/ml)			
≤5 vs >5	**<0.0001**	1.818	1.370-2.412
CA199 (U/ml)			
≤37 vs >37	**<0.0001**	1.694	1.279-2.243
CA724 (U/ml)			
≤6.9/>6.9	**0.009**	1.483	1.102-1.996
CA125(U/ml)			
≤35 vs >35	**0.001**	1.779	1.284-2.466
CA50			
≤25 vs >25	**<0.0001**	1.733	1.283-2.342
PD-L1			
Negative vs Positive	0.938	0.986	0.693-1.403
CD3+T cells			
Low vs High	0.946	0.989	0.725-1.351
CD4+T cells			
Low vs High	**0.026**	0.700	0.512-0.957
CD8+T cells			
Low vs High	**0.036**	0.715	0.523-0.978

The results with statistical differences are bold values.

**Table 6:** Multivariate Cox regression analysis of overall survival in gastric cancer patients.

Parameters	Multivariate COX regression analysis		
	*P* value	HR	(95%CI)
CD3D expression			
High vs Low	**0.046**	0.687	0.474-0.994
CD4+T cells			
Low vs High	**0.013**	0.617	0.421-0.903
CD8+T cells			
Low vs High	**<0.0001**	0.332	0.193-0.570
CD3+T cells			
Low vs High	**<0.0001**	3.485	1.911-6.355
PD-L1			
Negative vs Positive	0.602	1.104	0.760-1.604
Gender			
Female vs. Male	0.726	1.075	0.717-1.612
Age (year)			
≥65 vs <65	0.142	1.278	0.922-1.772
Gastric history			
Yes vs No	**0.005**	1.883	1.205-2.941
Smoking history			
Yes vs No	0.771	0.942	0.631-1.407
Drinking history			
Yes vs No	0.722	1.081	0.704-1.660
Weight loss			
Yes vs No	0.188	1.256	0.895-1.763
TNM stage			
I+II vs III+IV	**<0.0001**	2.787	1.640-4.736

The results with statistical differences are bold values.

**Figure 4:**
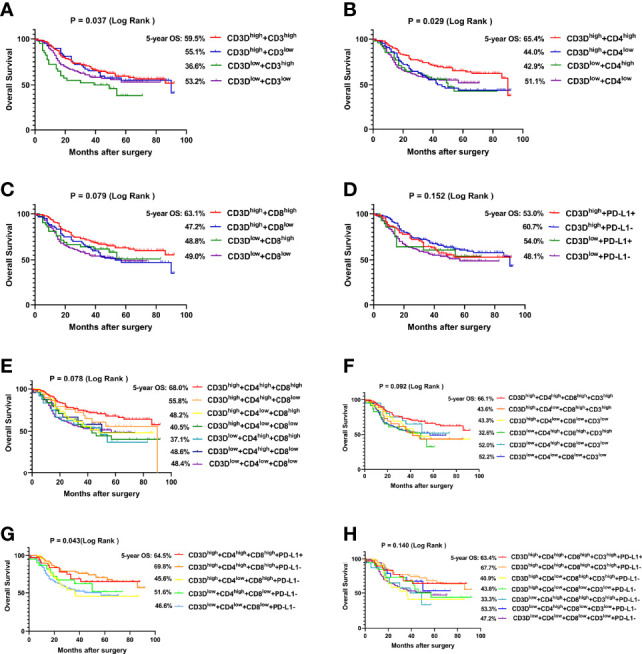
CD3D^high^ combined with CD4^high^, CD8^high^ and PD-L1- predicts the best prognosis of gastric cancer. **(A)** The overall survival (OS) curves of gastric cancer patients with different CD3D combined with CD3 expression levels in tumor tissues. **(B)** The OS curves of gastric cancer patients with different CD3D and CD4 expression levels in tumor tissues. **(C)** The OS curves of gastric cancer patients with different CD3D and CD8 expression levels in tumor tissues. **(D)** The OS curves of gastric cancer patients with different CD3D combined with PD-L1 expression levels in tumor tissues. **(E)** The OS curves of gastric cancer patients with different CD3D, CD4 and CD8 expression levels in tumor tissues. **(F)** The OS curves of gastric cancer patients with different CD3D, CD4, CD8 and CD3 expression levels in tumor tissues. **(G)** The OS curves of gastric cancer patients with different CD3D, CD4, CD8 and PD-L1 expression levels in tumor tissues. **(H)** The OS curves of gastric cancer patients with different CD3D, CD4, CD8, CD3 and PD-L1 expression levels in tumor tissues. E-H: Because of the large number of groups, we only show some groups with large number of cases.

Since CD3D was positively correlated with the expression of CD3, CD4, CD8 and PD-L1, the effect of CD3D in combination with these factors on the prognosis of gastric cancer was analyzed. As shown in **Figures 4A–D**, the 5-year overall survival rate of CD3D^high^ + CD3^high^ patients was 59.5%, that of CD3D^high^ + CD4^high^ patients was 65.4%, that of CD3D^high^ + CD8^high^ patients was 63.1%, and that of CD3D^high^ + PD-L1- was 60.7%, which was higher than all other groups. As shown in **Figures 4E–H**, the 5-year overall survival rate of CD3D^high^ + CD4^high^ + CD8^high^ patients was 68.0%, that of CD3D^high^ + CD4^high^ + CD8^high^ + CD3^high^ was 66.1%, that of CD3D^high^ + CD4^high^ + CD8^high^ + PD-L1- was 69.8%, and that of CD3D^high^ + CD4^high^ + CD8^high^ + CD3 ^high^ + PD-L1- was 67.7%. Thus, the combination of CD3D^high^ with CD4^high^, CD8 ^high^ and PD-L1- predicts the best prognosis of gastric cancer.

## Discussion

The exploration of gene expression profiles, the tumor mutational burden, PD-L1 and other biomarkers have yielded great progress in predicting patient prognosis and immunotherapy efficacy, but there are still some problems that make it difficult to meet clinical needs (18, 19). In addition to clarifying the role of TILs, a comprehensive understanding of the tumor immune microenvironment can help to guide individualized immunotherapy, making it a hot spot in cancer immunotherapy research (20). Multiple studies have shown that TILs are often associated with better treatment response and prognosis (21–23). However, due to the heterogeneity of gastric cancer and the complexity of the immune microenvironment, PD-L1-based immune checkpoint inhibitors have limited benefits in the treatment of gastric cancer (24). Moreover, TILs have also been found to play a limited role in the prognosis and efficacy prediction of gastric cancer. Therefore, it is urgent to find new targets to aid in diagnosis and in predicting prognosis and immunotherapy response to improve the diagnosis and treatment of gastric cancer patients.

CD3D is part of the T-cell receptor/CD3 complex (TCR/CD3 complex), and defects in this gene cause severe combined immunodeficiency (25). CD3D has been found to be associated with prognosis in various tumors. Shi et al. have demonstrated that the CD3D/CD4 ratio was a stable independent prognostic factor in muscle-invasive bladder cancer (26). In addition, other studies have shown that high CD3D expression is strongly associated with poor survival in breast carcinoma (27). In contrast, in patients with colorectal cancer, high CD3D expression was found to predict better clinical outcomes (28). In other words, CD3D may play completely opposite roles in different tumors. In our study, we found that CD3D was highly expressed in gastric cancer tissues compared with paracancerous tissues. Univariate and multivariate analyses showed that CD3D was an independent prognostic factor for gastric cancer, and patients with high expression of CD3D had a better prognosis. In addition, CD3D was negatively correlated with the Borrmann type and distant metastasis.

TILs refer to lymphocytes that leave the blood stream to enter the tumor, constituting an important part of the tumor microenvironment (29). They have been found to have an important role in predicting tumor prognosis and have even been employed as a means of cell therapy (30). The PD-1/PD-L1 signaling pathway has been shown to be important for tumor immunosuppression, inhibiting the activation of T lymphocytes and enhancing the immune tolerance of tumor cells, exhibiting an important regulatory role in the tumor immune microenvironment. Many studies have shown that the expression of TILs (CD3, CD4, CD8) is associated with a favorable prognosis in gastric cancer (31–33). However, the relationship between PD-L1 and gastric cancer prognosis is controversial. Some studies have shown that positive PD-L1 expression is significantly related to poor overall survival (34–36), but some studies have failed to confirm this finding (37, 38). In our study, we found that CD4 and CD8 were good prognostic factors for gastric cancer in both univariate and multivariate analyses. CD3 was not found to be associated with prognosis in the univariate analysis but was identified as a good prognostic factor for gastric cancer in the multivariate analysis. In contrast, PD-L1 was not associated with gastric cancer prognosis in either the univariate or multivariate analysis.

Previous studies have shown that CD3D can modulate the tumor immune microenvironment and affect the prognosis and immune response of breast cancer and cervical squamous cell carcinoma (39–41). In gastric cancer patients, we found that the expression of CD3D was highly positively correlated with the expression of CD3, CD4, CD8, and PD-L1. In addition, the combination of CD3D with CD3, CD4, CD8 and PD-L1 predicted the best prognosis, with the 5-year survival rate of the CD3D^high^ + CD4^high^ + CD8^high^ + PD-L1- group being the highest, reaching 69.8%. Thus, CD3D may play an important role in the regulation of the immune microenvironment in gastric cancer.

## Data Availability Statement

The original contributions presented in the study are included in the article/supplementary material. Further inquiries can be directed to the corresponding authors.

## Ethics Statement

The study was approved by the research ethics committee of Cancer Hospital of Zhejiang Cancer Hospital (IRB-2021-431). The patients/participants provided their written informed consent to participate in this study.

## Author Contributions

QY, BZ, and JQ conceived the study and acquired the funding. LY and JX carried out clinical research, collected clinical samples, analyzed clinical data, and wrote articles. YS, ZJ, ZB, YW, PY, and YX participated in clinical sample collection. All authors have read and approved the final manuscript.

## Funding

This study was supported by the National Key R&D Program of China (2021YFA0910100), Natural Science Foundation of Zhejiang Province (HDMY22H160008), Program of Zhejiang Provincial TCM Sci-tech Plan (2018ZY006), Medical Science and Technology Project of Zhejiang Province (2022KY114, WKJ-ZJ-2104), Zhejiang Provincial Research Center for Upper Gastrointestinal Tract Cancer (JBZX-202006), Science and Technology Projects of Zhejiang Province (2019C03049), and National Natural Science Foundation of China (82074245, 81973634).

## Conflict of Interest

The authors declare that the research was conducted in the absence of any commercial or financial relationships that could be construed as a potential conflict of interest.

## Publisher’s Note

All claims expressed in this article are solely those of the authors and do not necessarily represent those of their affiliated organizations, or those of the publisher, the editors and the reviewers. Any product that may be evaluated in this article, or claim that may be made by its manufacturer, is not guaranteed or endorsed by the publisher.
